# Endoscopic Assessment and Serial Balloon Dilatation in a Toddler With Dyskeratosis Congenita-Hoyeraal-Hreidarsson Syndrome Following Bone Marrow Transplant: A Case Report

**DOI:** 10.1097/PG9.0000000000000291

**Published:** 2023-03-13

**Authors:** Kurt Rodriguez, Ryan Shargo, Morgan Ekblad, Gauri Sunkersett, Sara Karjoo, Marisol Betensky, Michael J. Wilsey

**Affiliations:** From the * Johns Hopkins All Children’s Hospital, Saint Petersburg, FL; † University of South Florida, Reisterstown, MD; ‡ AbbVie, South San Francisco, CA.

**Keywords:** gastroenterology, esophagus, stricture, dyskeratosis, congenital, allogenic, bone, marrow, transplant, pediatrics, dilation

## Abstract

We report a 3-year-old patient with suspected oropharyngeal graft-versus-host disease (GVHD) who developed progressive dysphagia to solids and liquids. The patient has a history of Dyskeratosis Congenita-Hoyeraal-Hreidarsson Syndrome with associated bone marrow failure requiring a nonmyeloablative matched sibling hematopoietic stem cell transplant. Esophagram revealed significant narrowing in the cricopharyngeal region. Subsequent esophagoscopy showed a proximal, high-grade pinhole esophageal stricture that was very difficult to visualize and cannulate. High-grade esophageal strictures are uncommon in very young children with GVHD. We believe the patient’s underlying Dyskeratosis Congenita-Hoyeraal-Hreidarsson Syndrome in the setting of inflammatory changes seen in GVHD following hematopoietic stem cell transplant set the stage for a high-grade esophageal obstruction. The patient’s symptoms improved with serial endoscopic balloon dilation.

## INTRODUCTION

Dyskeratosis congenita (DC) is a short telomere syndrome resulting from germline mutations in telomere maintenance genes characterized by mucocutaneous manifestations, progressive bone marrow failure, and increased cancer risk (1–3). Hoyeraal-Hreidarsson syndrome (HHS) is considered a clinically severe variant of DC that presents early in childhood with a constellation of symptoms, including intrauterine growth retardation (IUGR), cerebral hypoplasia, immunodeficiency, and progressive bone marrow failure (4). DC-HHS treatment includes allogeneic hematopoietic stem cell transplant (HSCT). Although bone marrow failure and immunodeficiency are effectively treated with HSCT, patients with DC-HHS remain at risk for other extra-bone marrow manifestations. Gastrointestinal manifestations, including feeding difficulties and digestive tract abnormalities, have been reported in DC-HHS patients (2). However, there are few reports of esophageal strictures in young children (3–5), and this complication occurs more commonly in older children and adults (1–3,6).

Immunocompetent donor-derived stem cells can mount an immune response targeting the recipient’s tissue antigens, leading to graft-versus-host disease (GVHD). GVHD primarily manifests as skin, liver, and gastrointestinal tissue involvement (1). GVHD occurs uncommonly in the esophagus and presents as desquamative esophagitis, esophageal casts, ulcerations, webs, and rarely strictures (7,8). High-grade esophageal strictures from GVHD in very young children have not been widely reported. We consider the subsequent inflammatory changes corresponding with GVHD plausibly complicated the pretense of DC-HHS following HSCT. The patient’s dysphagia symptoms and ability to tolerate oral secretions significantly improved with serial endoscopic balloon dilation.

## CASE PRESENTATION

The patient is born prematurely at 29 weeks gestation with IUGR, pontocerebellar hypoplasia, microcephaly, developmental delay, failure to thrive, and feeding difficulties. The patient underwent a percutaneous endoscopic gastrostomy tube placement for nutritional support at 19 months of age, at which time the esophagus was endoscopically normal. Shortly afterward, she developed thrombocytopenia, and a bone marrow failure work-up revealed abnormally short telomeres and 2 heterozygous *RTEL1* mutations confirming the diagnosis of DC-HHS. The patient developed progressive bone marrow failure that required nonmyeloablative HLA-matched sibling HSCT at 33 months of age. Her posttransplant course was complicated by the development of chronic skin and liver GVHD starting 6 months posttransplant, which required several modalities of immunosuppressive therapy, including topical creams, oral steroids, Rapamycin, and Mycophenolate. She developed symptoms consistent with oropharyngeal GVHD, for which she was treated with a 10-day course of dexamethasone mouthwash.

**FIGURE 1.**
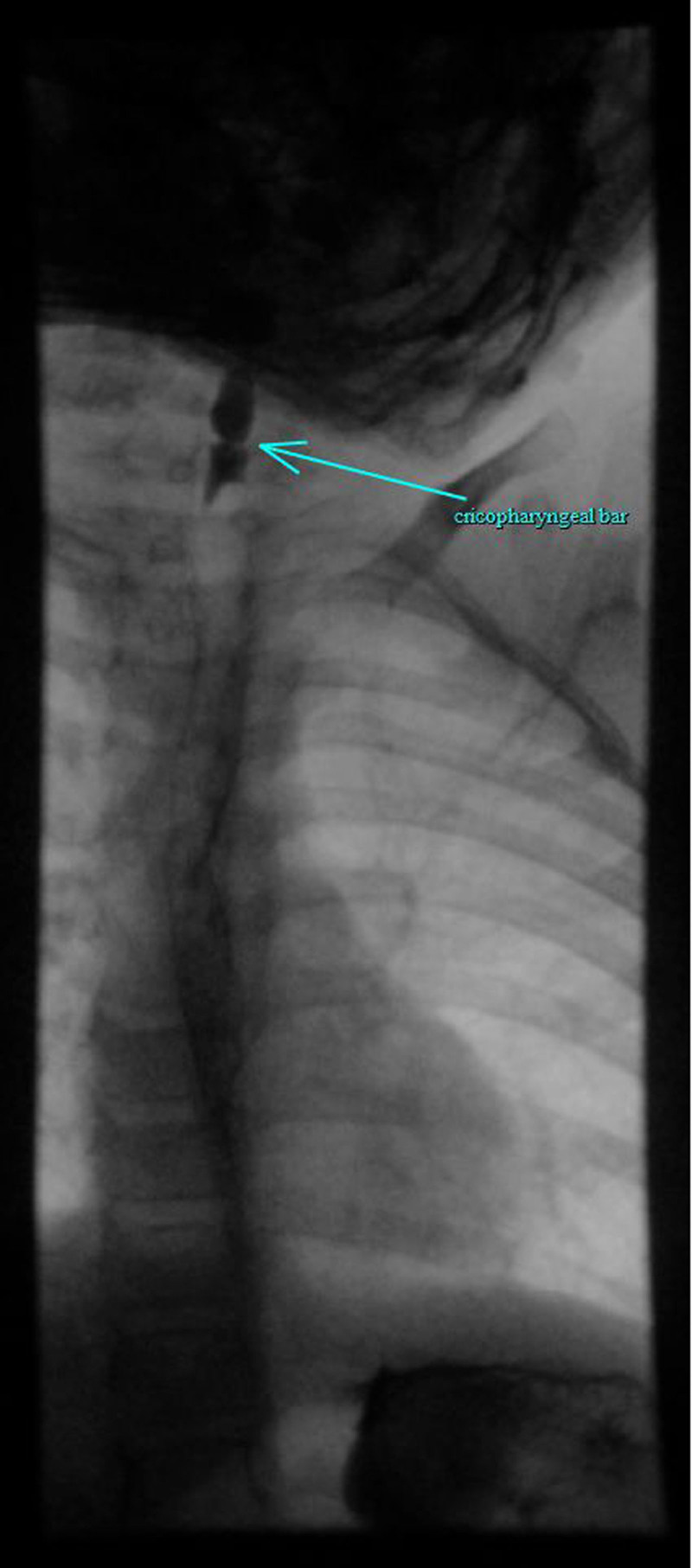
Esophageal bar in the cricopharyngeal region.

**FIGURE 2.**
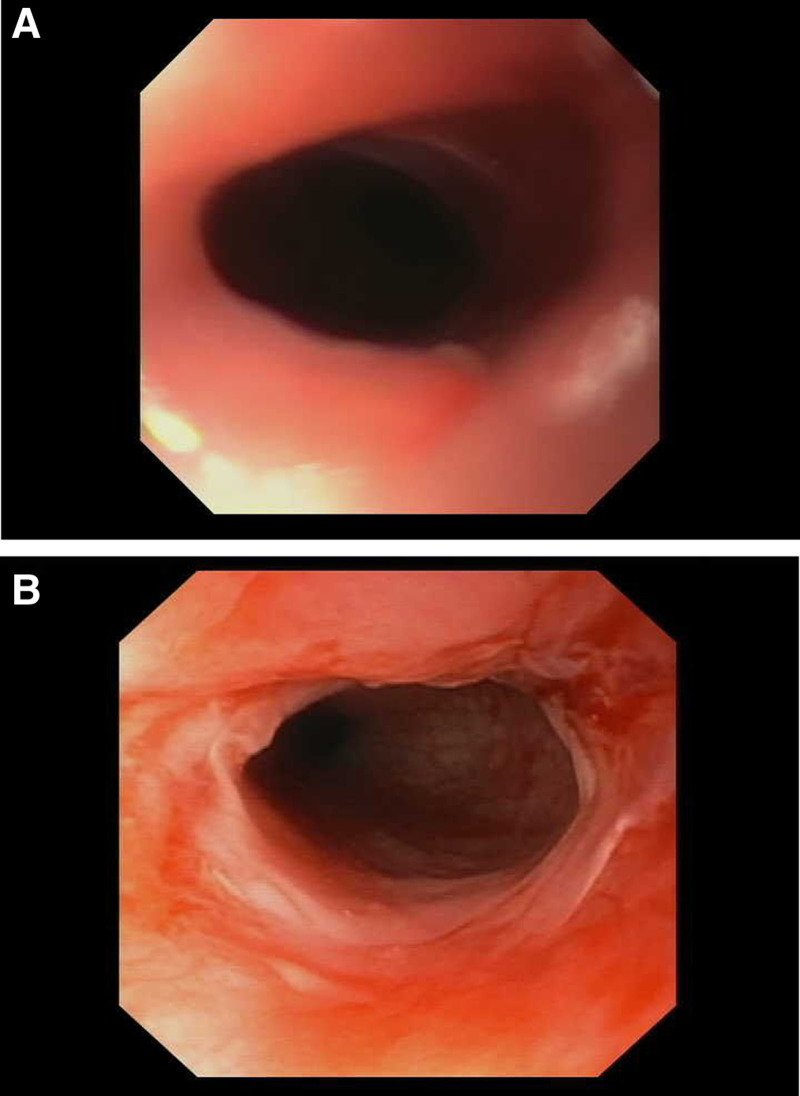
High-grade proximal esophageal stricture. A) Stricture approximately 2 mm in diameter, successfully dilated to 6 mm. B) Status postdilation to 10 mm.

The patient presented at 3.5 years of age (9 months posttransplant) with symptoms of progressive dysphagia, drooling, and vomiting with solid foods. Before this, the patient drank water well and ate bites of food but needed supplemental gastrostomy feeding. A contrast esophagram revealed a significantly narrowed upper esophagus in the cricopharyngeal region (Fig. 1). Subsequent esophagogastroduodenoscopy showed an extremely proximal, high-grade pinhole esophageal stricture that was difficult to visualize and even more difficult to cannulate (Fig. 2). After many attempts, guidewire exploration successfully cannulated the pinhole stricture under endoscopic and fluoroscopic guidance. A 6-7-8-mm balloon dilatation catheter was backloaded onto the wire, and the esophageal stricture was slowly dilated up to 6 mm and held in place for 2 minutes. There was some bleeding noted but no signs of perforation on fluoroscopy. The stricture was injected with intralesional steroids (1 mg/kg of triamcinolone acetonide divided into four aliquots) to help decrease the potential for postdilation inflammation. The patient’s esophageal stricture was successfully dilated 2 weeks later to 8 mm, then 1 month later to 10 mm, each with steroid injection and a significant improvement of her dysphagia symptoms (dilations were eventually halted due to a spike in the COVID-19 pandemic). At her telemedicine follow-up 3 months postprocedure, the patient was back to baseline, reportedly happy and playful tolerating bites of food and liquids by mouth without drooling or vomiting.

## DISCUSSION

Gastrointestinal manifestations are not uncommon in patients with short telomere syndromes such as DC. They can be one of the presenting and most difficult symptoms to treat, particularly in patients with the clinically severe variant DC-HHS. Although the underlying mechanisms are not fully understood, short telomere length disrupts the gastrointestinal (GI) mucosal integrity, leading to a broad spectrum of GI symptoms ranging from mild feeding difficulties to life-threatening severe enteropathy/enterocolitis requiring colectomy and total parenteral nutrition (3). Esophageal strictures occur more frequently in adults with DC and are present in approximately 25% of DC cases (3,6). There are few reports of DC esophageal strictures in young children (3–5). Jonassaint et al (3) reported a 3-year-old boy with DC and dysphagia was discovered to have a proximal esophageal web. Sawant et al (5) reported an 8-year-old boy with DC whose dysphagia symptoms resolved after esophageal dilation for distal esophageal stricture.

Esophageal strictures can also occur in patients with GVHD following HSCT (7,9–11). McDonald et al (9) reported the radiographic features of esophageal involvement and chronic GVHD of 105 transplant patients with chronic GVHD. During 5 years, 14 developed esophageal symptoms in addition to radiographic and endoscopic findings, with a mean age of 25.7 years (range 12 to 50 years) (9). Today, GVHD appears less common in patients receiving a nonmyeloablative conditioning regimen, with a cumulative incidence of acute GVHD (grade II-IV) at 18% and chronic GVHD at 31% (12). While GVHD commonly affects the skin, liver, and gastrointestinal tract, high-grade esophageal strictures are uncommon in young children.

There are few reports of DC-HHS patients experiencing esophageal strictures in the setting of GVHD after HSCT (13). Ghavamzadeh et al reported 2 young adult siblings (ages 18 and 21) with DC who underwent HSCT with complications of GVHD and esophageal strictures (13). We believe our patient’s underlying DC-HHS in the setting of inflammatory changes seen in GVHD following HSCT accelerated the formation of a high-grade esophageal stricture in this young child with a previously normal esophagus before HSCT. Histologic diagnosis rather than a suspected or clinical diagnosis of GVHD is ideal, as both short telomere syndromes and GVHD may potentially have similar effects on cells and can be difficult to distinguish (13). However, esophageal tissue was not obtained in this patient before the cessation of esophageal dilation therapy.

## CONCLUSION

Herein, we report a case of high-grade esophageal stricture in a toddler with DC-HHS following HSCT complicated by suspected GVHD. Early recognition of this complication is imperative for clinicians caring for patients with this rare disease. Endoscopic assessment is feasible and esophageal strictures are amenable to serial endoscopic balloon dilatation. This case highlights the need for early evaluation of at-risk toddlers with DC-HHS and/or GVHD presenting with dysphagia and feeding difficulties.

## ACKNOWLEDGMENTS

Informed patient consent was obtained from the parents for publication of the details of this case.
